# Cultural Roots of Parenting: Mothers’ Parental Social Cognitions and Practices From Western US and Shanghai/China

**DOI:** 10.3389/fpsyg.2021.565040

**Published:** 2021-04-13

**Authors:** Huihua He, Satoshi Usami, Yuuki Rikimaru, Lu Jiang

**Affiliations:** ^1^Department of Early Childhood Education, College of Education, Shanghai Normal University, Shanghai, China; ^2^Graduate School of Education, University of Tokyo, Tokyo, Japan; ^3^Center for Research and Development on Transition From Secondary to Higher Education, University of Tokyo, Tokyo, Japan

**Keywords:** cultural values, social cognitions, parenting practices, European American, Shanghai/China

## Abstract

Cultural values can be considered as important factors that impact parents’ social cognitions and parenting practices. However, few studies compare specific cultural values of parents and the relationships between cultural values and parenting processes in eastern and western contexts. This study examined the ethnicity differences in mothers’ cultural values, parental social cognitions (child-rearing ideologies and goals), and parenting practices between Mainland Chinese and European American contexts. Predictors of parenting goals and parenting practices were also investigated. Mothers of 4–6 years old children from the western United States (*N* = 78) and Shanghai/China (*N* = 96) participated in this study. The results suggested that mothers from Shanghai/China were both more collectivistic and individualistic than mothers from the western United States. Chinese mothers more strongly endorsed training and collectivistic parenting goals, while European American mothers more strongly endorsed individualistic parenting goals for their children. However, no significant difference was found in parenting practices for both groups of mothers. For both ethnic groups, in general, mothers’ cultural values have small but significant impact on their parenting processes. The prediction of cultural values and parenting goals on parenting practices were also different for both ethnicity groups. Although Chinese mothers were higher on both individualism and collectivism, their collectivistic values were more important in predicting parental social cognitions.

## Introduction

Ecological theory suggests that human development involves progressive and mutual accommodation between an active, growing human being and the changing properties of the environment in which the developing individual lives (Bronfenbrenner, 1979). As such, the specific contexts or environments in which children are raised influence their concurrent outcomes as well as their subsequent development. Parents with different cultural values could be assumed to have different parental values and parenting practices. In this perspective, examining parental values and behaviors requires one to be cognizant of how environmental factors, such as culture, impact and provide a source of parents’ child-rearing ideologies and parenting goals and practices.

According to O’Hagan (1999), culture is the distinctive way of life of the group, race, class, community, or nation to which the individual belongs. It is the product of the values and perceptions that constitute the individual’s knowledge and understanding of the world. According to the value theory that was proposed by Schwartz, values could be used to characterize cultural groups, societies, and individuals and to explain the motivation of human behaviors (Schwartz, 1990). Values are beliefs linked to affect; it could refer to desirable goals, could serve as standards or criteria and ordered by importance. More importantly, values could guide human actions. So cultural values shape an individual’s perspectives of the world and permeate every aspect of daily life. As such, cultural values can affect individuals directly by establishing cultural expectations regarding people’s dress, their customs, their patterns of work, religious ceremonies, and leisure pursuits (Giddens, 1993). Indirectly, cultural values provide norms or expectations for such things as education and childrearing.

An increasingly common way to conceptualize cultural value is to focus on the constructs of collectivism and individualism from the 1980s (Hofstede, 1983). Individualism refers to the extent to which individuals consider themselves as independent from others in their social or cultural group. Individuals who hold more individualistic cultural values are said to be motivated by their own preferences, goals, and benefits (Hofstede, 1983; Triandis, 1995). Alternatively, collectivism is defined as the extent to which individuals consider themselves as being related to or connected with others with such individuals expressly motivated by the norms and values of their cultural or social group (Mann and Cheng, 2013). Individuals who prioritize more collectivistic cultural values are said to be motivated by the norms and values of their social or cultural group (Markus and Kitayama, 1991; Mann and Cheng, 2013).

Dichotomy of individualism/collectivism is one of the most useful and widely researched constructs for understanding cultural variation in parents’ beliefs and practices (Schwartz, 1990; Triandis, 1995; Cote and Bornstein, 2003). Conceptually, individualism and collectivism map globally to Eastern and Western cultures, which differ in terms of history, values, and beliefs. American culture has been described as traditionally individualistic, in that it conceives the individual as an independent and autonomous entity comprising a unique configuration of internal attributes and behaves primarily as a consequence of those internal attributes (Oyserman et al., 2002; Huntsinger and Jose, 2009). Conversely, many Eastern cultures, particularly Asian countries, such as China, are described as having a collectivistic cultural value. These individuals are said to be interdependent in that they emphasize the fundamental connections of human beings to one another. Whereas EA parents often see the need to encourage their child to be independent and unique, Chinese parents are more likely to encourage children to view themselves as part of the integrated whole of their family, community, and society, and not to emphasize their differences from others (Markus and Kitayama, 1991). As such, American children may learn to see the world strictly on an individual basis, whereas Chinese children may learn to see the world as a network of relationships.

Although dichotomization of cultural values (collectivism vs. individualism) is a useful conceptual framework for understanding parental values, goals, and practices, researchers suggest that caution should be taken when considering similarities and differences between parents from different cultures (Bornstein, 2012; Cheah, 2016). This caution arises from the fact that cultures are not homogeneous entities without individual variation (Bornstein, 2012). It would not be appropriate, for example, to assume that individualistic cultures lack a concept of relatedness and that collectivistic cultures fail to recognize the concept of individual choice.

The relation between collectivistic and individualistic cultural values has been highlighted by Oyserman et al. (2002). In their meta-analysis of 50 existing individualism and collectivism studies, Oyserman et al. (2002) found that individualism and collectivism are not opposing constructs. Rather, they appear to be statistically independent or orthogonal in nature. Hence, an individual doesn’t necessarily have to be low in one dimension in order to be high in the other. Their findings also demonstrate that individuals in some cultures that have traditionally been considered to be primarily collectivistic (e.g., South Korea) or individualistic (e.g., Australia) may in actuality be less so. Given the results of their meta-analysis, it is important to consider individualism and collectivism to be multifaceted dimensions that may coexist within a given culture. As such, they may be useful in describing differences and similarities among ethnic groups as well as providing a meaningful way to tie parenting beliefs and practices to a larger cultural context.

One of the most important tasks of parenting is to help children achieve competence in a specific cultural context (Cheah and Rubin, 2003; Bornstein, 2012). During the last decade, cross-cultural parenting research has reflected an increasing awareness and recognition that much of parental cognition and practices are culturally organized (Meng, 2012; Cheah, 2016). In particular, several researchers have investigated cultural differences in parental values, goals, and practices (Cheah et al., 2013, 2015; Khaleque, 2013). These studies suggest that parental social cognitions and practices need to be understood and studied within the cultural contexts in which they occur.

For example, parental values can be defined as ideologies that are statements of truth from the parents’ point of view and they encompass parents’ cognitions and beliefs about raising children and being a parent (Sigel, 1985; McGillicuddy-De Lisi and Sigel, 1995). In a previous study, Asian-American parents were reported to place a heavy emphasis on the importance of achievement in childhood (Okagaki and Frensch, 1998). Such views are most likely a reflection of parents’ cultural values and are important when considering the impact that parenting beliefs and goals have on children’s developmental outcomes.

Parental goals refer to the specific characteristics or traits that parents try to encourage or discourage in their children through specific childrearing interactions. Cultural values are tied to parents’ conceptualization of parenting goals. According to Levine (1988), the ways in which parenting goals are operationalized depend on the family’s social, cultural, and economic context. For example, Miller et al. (2002) concluded that for American parents, self-esteem is a central goal of parenting, while Chinese parents have higher expectations for their children’s educational attainments (Okagaki and Frensch, 1998). In addition, Gorman (1998) also suggest that immigrant Chinese mothers characterize their parenting goals in relation to their cultural values, such as being self-sufficient, fulfilling one’s obligations, respecting elders, and caring for others.

Parenting practices have been defined as a wide range of ways that parents elicit, inhibit, influence, or otherwise control their children’s behavior during day-to-day interactions. They are more specific ways that parents address their values and achieve their goals (Power and Manire, 1992). The majority of research on parenting practices has almost exclusively focused on parenting style in both Chinese and American contexts. In the Western context, research on the dimensions of warmth and control has yielded a comprehensive model of four types of parenting styles: authoritarian, authoritative, indulgent, and indifferent-uninvolved (Baumrind, 1971). Chinese parents were found to be more authoritarian than American parents (Chao, 1994, 2000). Chao also suggested that understanding how Chinese parents’ “train” their children puts into doubt the conceptual usefulness of parental demand and responsiveness for studying Chinese populations. Training can be defined as “a continuous monitoring and guidance of children” (Chao, 2000, p. 234). She suggests that training is an essential Chinese parent form of nurturing. The nurturing aspect of training is reflected in parental involvement and support, but it does not include overt demonstrations of the parents’ affection for the child, which are often emphasized by Western cultures.

Studying parents’ cultural values is useful because it provides us with an orienting tool for understanding the development and organization of parents’ social cognitions and parenting behaviors. For example, in terms of parental goals, Chinese American and Taiwan Chinese parents were found to rate collectivistic parental goals more important than European American parents. In particular, Chinese parents rated persistence, politeness, concentration, and precision as more valuable than did European American mothers (Jose et al., 2000). In addition, recently Cheah et al. (2015) found the differences in Chinese immigrant and European-American mothers’ expressions of warmth toward their preschool children. They found that Chinese immigrant mothers expressed less warmth than European-American mothers. Cultural differences were also found to interpret these different parental behaviors. European American mothers focused on more direct and outward demonstrations of warmth based on Western cultural values. Moreover, Chen et al. (2014) also found that cultural values were highly related to parents’ self-reported expressivity. Eastern cultural values were generally associated with less emotional expression in the family. Hence, according to previous studies, it is concluded that European American parents who emphasized a more individualistic values reported different parental goals and practices from parents who emphasized more collectivistic cultural values.

To date, however, although many cross-cultural studies have focused on examining ethnic differences on parenting values, goals, and practices, most of them fail to directly assess parents’ endorsement of cultural values. Rather, many of these studies examine parental cognitions and practices and then, upon finding differences in parental values or practices across different ethnic groups, use culture as a *post hoc* explanation for why parents differ. Two problems exist with this approach. First, researchers may overgeneralize the effect that cultural values have on parental cognitions and practices. Falling back on culture as an explanation for differences may in actuality inflate differences between groups.

Second, approaches that compare parents from different cultures and then explain findings by making summary statements about cultural values may limit our understanding of culture and parenting processes. Essentially, they may ignore the fact that as much variation exists within cultures as between (Triandis and Gelfland, 1998; Cote and Bornstein, 2003; Camras et al., 2012). As such, instead of only conducting inter-cultural comparisons between Western and Eastern parenting, intracultural comparisons should also be considered as valuable methods to reveal variation in parenting processes within one cultural context. The current study would discuss the multifaceted nature of collectivistic and individualistic values that can coexist in one culture.

Therefore, the first goal of the present study was to examine ethnic differences in collectivistic and individualistic cultural values, child-rearing ideologies, parenting goals, and parenting practices between mothers from the western United States and Shanghai/China. We hypothesized that mothers from Shanghai/China would highly endorse collectivistic cultural values, collectivistic parenting goals, and child-rearing ideologies about training while mothers from the western United States would have higher scores in individualistic cultural values, individualistic parenting goals, and practices in nurturance.

In addition, child gender has been considered as a peripheral variable in studies of parental values, goals, and practices (Cheah and Rubin, 2004; Park and Cheah, 2005; Schönpflug and Yan, 2013). For example, mothers of boys reported different parental attributions of young children’s social behaviors from mothers of girls (Endendijk et al., 2016). A recent study also argued that gendered socialization is rarely founded as statistically significant in broad parenting style and parenting practices, and it could be an implicit variable so that parents could offer different products and responding to children differently according to children’s gender (Schönpflug and Yan, 2013). Given that, gender effect and interaction effect between gender and ethnicity would also be hypothesized.

The second purpose of the study was to investigate relationships among cultural values and parenting for mothers from the western United States and Shanghai/China, respectively. Pearson product-moment correlations would be performed to explore difference patterns of correlations for two groups of mothers. We hypothesized that significant correlations among cultural values and parenting for each ethnical group of mothers would be hypothesized.

The third purpose of the study was to examine the effects of cultural values on parental values and goals and practices for each group of mothers, respectively. We hypothesized that mothers’ cultural values that mothers endorsed would predict their parental values and goals. We also expected that for both ethnic groups of mothers, cultural values would interfere with parenting practices and their parental values and goals would also contribute to the variance of parenting practices.

## Materials and Methods

### Participants

One hundred and seventy-four mothers of 4–6 years old children were recruited for this study. Ninety-six mothers were recruited from Shanghai, China, while 78 European American mothers (European Americans means Americans of European ancestry) were recruited from the state of Washington in the United States. Fifty of the 96 Mainland Chinese children were female (52.1%) while 41 of the European American children were female (52.6%). The mean age for Mainland Chinese children was 4.85 (*SD* = 0.79) years old and for European American children was 4.71 (*SD* = 0.99) years old.

In general, mothers reported high education attainment. Using the same scale for each group (i.e., 1 = some college or lower, 2 = college, and 3 = higher than college), most Mainland Chinese and European American mothers reported that they had college degrees (see Table 1). Both European American and Mainland Chinese mothers were asked to report their monthly income by choosing one item of 3-point scale (i.e., 1 = $3,000 or lower, 2 = $2,000–$3,000, 3 = more than $6,000). In the questionnaire for Mainland Chinese mother, Chinese yuan currency unit was used. The amount of Chinese yuan that Chinese mothers reported was transformed to dollars.

**TABLE 1:** Demographic characteristics of mothers and children.

**Demographic characteristics**	**Shanghai/China**		**Western US**	
	**N**	**%**	**N**	**%**
**Children’s gender**				
Boys	46	47.9	37	47.4
Girls	50	52.1	41	52.6
**Mother’s education level**				
Some college or lower	24	25.0	18	23.1
College	39	40.6	32	41.0
Higher than college	33	34.4	28	35.9
**Mother’s monthly income**				
3,000 or lower	37	38.4	30	38.5
3,000–$6,000	28	29.2	22	28.2
More than $6,000	31	32.4	26	33.3

It is difficult to compare family income precisely because American dollars and Chinese yuan use quite a different scale and currency. Also, the purchase price of merchandise in each country is also quite different. Participants from Mainland China were mothers living or working in Shanghai. According to Shanghai Statistical Yearbook, Shanghai Municipal Bureau of Statistics (2019), average income per month was 15,000–20,000 Chinese yuan (2,100–2,800 US dollars). Moreover, according to the demographic characteristic of mothers participating in our study, average income per month of most EA and MC mothers were above 3,000 US dollars. Given this, both groups of mothers were from the middle class of the area they were living and working.

### Measures

Three sets of questionnaires were employed to measure mothers’ cultural values, child-rearing ideologies and goals, and parental practices.

#### Cultural Values

The Individualism/Collectivism Scale (Singelis et al., 1995) was employed to assess mothers’ collectivistic and individualistic cultural values. This measure has two subscales asking participants to report on a 7-point Likert-type scale, from 1 (*strongly disagree*) to 7 (*strongly agree*), how much they agree with 32 statements reflecting individualism (16 items) and collectivism values (16 items). Examples of individualistic cultural values are “Without competition it is not possible to have a good society” and “Being a unique individual is important to me.” Examples of collectivistic cultural values include “It is my duty to take care of my family, even when I have to sacrifice what I want.” and “To me, pleasure is spending time with others.” Previous research has established the construct validity and internal consistency of this scale (Triandis, 1995; Mann and Cheng, 2013).

In this study, the reliabilities of the scales for European American (for individualism subscale α = 0.66, for collectivism subscale α = 0.69) and the Mainland Chinese (for individualism subscale α = 0.83, for collectivism subscale α = 0.69) sample may not as good as previous studies but were acceptable.

#### Parental Values and Goals

Mothers’ parental values were assessed by Child-Rearing Ideologies Questionnaire (CRIQ) (Chao, 1994). This 13-item scale covered two areas, “ideologies on child development and learning” (7 items) and “ideologies of the mother-child relationship” (6 items). Respondents need to indicate the degree to which they agree or disagree with each item on a seven-point scale, from 1 = *strongly disagree* to 7 = *strongly agree*. Sample items from the ideologies of child development and learning subscale are “Children are by nature born innocent,” “Mothers must begin training child as soon as ready,” and “Children can improve in almost anything if they work hard.” Sample items from the ideologies of the mother-child relationship subscale are “mothers primarily express love by helping child succeed, especially, in school” and “a child should be in the constant care of their mothers or family.”

Cross-cultural studies of parenting measured parental values by CRIQ for Taiwan Chinese (α = 0.72) and Chinese American parents (α = 0.71) and reported acceptable internal consistency levels for these ethnicity groups (Chao, 1994; Jose et al., 2000). Few studies explored European American parents’ parental values by using this method. Jose et al. (2000) reported total scores of two subscales and found that CRIQ yield unacceptable internal consistency (α = 0.46) but acceptable test-retest reliability (*r* = 0.61) for European American parents of young children (5–6 years old). However, as this is the most appropriate scale measuring parental values relating to collectivistic and individualistic cultural values, same scoring methods were used for the current study. The internal consistency level of the current study for both European American (α = 0.68) and Mainland Chinese mothers (α = 0.65) are also acceptable.

The Importance of Collectivist and Individualist Traits in Child Scale (Jose et al., 2000) was used to assess mothers’ parenting goals concerning their child’s desired personality traits. In response to the question “How important do you think it is to encourage the following personality traits in your child?” parents rated 5 individualist traits (e.g., sociability, creativity, self-confidence, and independence) and 7 collectivist traits (e.g., persistence, obedience, politeness, concentration, respect, and precision) by a seven-point scale (1 = *not at all important* to 7 = *extremely important*). Parents received an average score for the two clusters separately, a high score for a cluster indicated that the parent highly endorses that particular cultural value for their child. According to previous research focusing on European American parents and Chinese American parents, the internal consistencies of these two subscales are acceptable (α = 0.76 and 0.78; Jose et al., 2000).

In this study, the reliabilities of both individualistic traits and collectivistic traits for European American (α = 0.80 for collectivistic traits and α = 0.62 for individualistic traits) and Mainland Chinese sample were also acceptable (α = 0.75 for collectivistic traits and α = 0.76 for individualistic traits).

#### Parenting Practices

The Parenting Dimensions Inventory Short Version (PDI-S; Slater and Power, 1987) was used to assess mothers’ parenting practices. The PDI-S has been used in research on immigrant Chinese parents and was translated into Chinese by Kelly and Tseng (1992). In previous research conducted with immigrant Chinese and European American mothers, Sebire et al. (2016) reported Cronbach’s alphas of 0.87 and 0.76, respectively.

The first section of this scale was utilized for the present study. It was a 13-item 6-point Likert-type scale assessing parenting nurturance (6 items), inconsistency (4 items), and discipline (3 items). A sample item for nurturance could be “I encourage my child to talk about his or her troubles.” However, Cronbach’s alphas of inconsistency (α = 0.42 for Mainland Chinese parents, α = 0.61 for European American parents) and discipline (α = 0.55 for Mainland Chinese parents, α = 0.60 for European American parents) subscales were not acceptable for both ethnic groups. So only nurturance subscale (α = 0.68 for Mainland Chinese parents, α = 0.79 for European American parents) was used in further analysis.

### Procedures

The Institutional Review Board (IRB) at Washington State University (WSU) first approved this research project to collect data from parents of 4–6 years old children in both the United States and Mainland China. After that, mothers from the western United States and Shanghai/China were recruited from local preschool and kindergarten programs. Principals and program directors were contacted initially in person, and the protocol was explained to principals by the researchers in some depth. A letter inviting mothers to participate and a consent form were distributed through children’s preschool or kindergarten programs. Mothers who agreed to participate in this study signed the consent form, completed a questionnaire packet, and returned these to their child’s preschool or kindergarten teacher. Both groups of mothers were asked to respond to questions regarding their cultural values, parenting beliefs, and parenting practices. After completed questionnaires were obtained, mothers were sent a thank-you letter and a small gift.

The majority of questionnaires were initially developed in English. Mothers in Washington were distributed questionnaires in English while mothers in Shanghai were given questionnaires that had been translated into Chinese. Some questionnaires were previously translated into Chinese by other researchers, such as ICITCS and PDI. For questionnaires that have not been previously translated into Chinese, such as the Individualism/Collectivism Scale (Triandis, 1995), a forward and then back translation method were utilized. In this method, the researcher translated the questionnaires into Chinese and a native Chinese speaker back-translated the questionnaires into English. This method of forward and back translation has been shown to be an acceptable method for ensuring that questionnaires are translated correctly across languages (Kelly and Tseng, 1992).

## Ethical Statement

This study was approved by the Research Ethics Committee of Washington State University, Washington. All participants involved in our study were given written, informed consent forms, which were signed by each participant and their guardians and returned to us. The ethics committee approved this consent procedure.

## Results

### Ethnicity Differences in Cultural Values and Parenting

To explore the differences in cultural values, parental social cognitions, and parental practices between two groups of mothers, MANOVA were performed. Ethnicity and children’s gender served as independent variables while cultural values, parental social cognitions, and parental practices were served as dependent variables.

Descriptive statistics of study variables and results of ethnicity differences are presented in Tables 2, 3. Results indicated significant overall ethnicity differences between each group, *F*(6, 165) = 19.46, *p* < 0.001, *η^2^* = 0.41. No significant differences were found in children’s gender or interaction effects between ethnicity [*F*(6, 165) = 1.12, *p* > 0.05, *η^2^* = 0.04] and children’s gender [*F*(6, 165) = 0.91, *p* > 0.05, *η^2^* = 0.03].

**TABLE 2:** Descriptive statistics of mothers’ cultural values, child-rearing ideologies, parenting goals and practices.

**Groups**	**Collectivistic cultural values**		**Individualistic cultural values**		**Child-rearing ideologies**		**Collectivistic parental goals**		**Individualistic parental goals**		**Parenting practices**	
	***M***	***SD***	***M***	***SD***	***M***	***SD***	***M***	***SD***	***M***	***SD***	***M***	***SD***
Shanghai/China	5.45	0.60	4.65	0.56	4.89	0.50	5.95	0.69	5.44	0.81	5.14	0.56
Boys	5.43	0.47	4.45	0.56	4.84	0.44	5.94	0.70	4.47	0.77	5.02	0.30
Girls	5.46	0.67	4.61	0.50	4.95	0.48	5.92	0.65	5.42	0.84	5.27	0.53
Western US	5.11	0.60	4.32	0.53	4.23	0.72	5.12	0.86	5.83	0.66	5.26	0.60
Boys	5.13	0.59	4.26	0.47	4.26	0.67	5.40	0.85	5.86	0.70	5.21	0.60
Girls	5.10	0.61	4.09	0.63	4.32	0.70	5.37	0.74	5.82	0.62	5.30	0.60

**TABLE 3:** Ethnicity and gender differences in mothers’ cultural values, child-rearing ideologies, parenting goals and practices.

**Variables**	**Collectivistic cultural values**		**Individualistic cultural values**		**Child-rearing ideologies**		**Collectivistic parental goals**		**Individualistic parental goals**		**Parenting practices**	
	***F***	***η*^2^**	***F***	***η*^2^**	***F***	***η*^2^**	***F***	***η*^2^**	***F***	***η*^2^**	***F***	***η*^2^**
Ethnicity	13.87***	0.08	18.44***	0.10	48.09***	0.22	23.89***	0.12	11.75***	0.07	1.47	0.01
Gender	0.001	0.001	0.001	0.001	0.91	0.01	0.07	0.001	0.18	0.001	3.74	0.02
Ethnicity X Gender	0.14	0.001	3.89	0.02	0.05	0.001	0.001	0.001	0.01	0.001	0.73	0.004

***p < 0.001.

Specifically, first, mothers from Shanghai/China reported themselves as both more collectivistic and individualistic than European American mothers. Second, mothers from Shanghai/China held beliefs about the importance of training and collectivistic goals more strongly than mothers from the western United States. In contrast, mothers from the western United States more strongly endorsed individualistic goals to be important for their child. No significant difference was found between European American and Mainland Chinese mothers in terms of their parenting practices, suggesting that Mainland Chinese mothers have similar practices in nurturance with European American mothers on average.

### Cultural Variations in Relationships Between Cultural Values and Parenting

In order to examine how mothers’ cultural values predict their child-rearing ideologies and goals, and how cultural values and parenting social cognitions predict their parenting practices, Pearson product-moment correlations were first calculated. Since results of multivariate analysis of variance indicated strong ethnicity differences between two ethnicity groups, correlation analysis was conducted separately for mothers from two different ethnicity groups to examine the pattern of relationships among the variables of interest. Second, a number of multiple regression analyses were performed separately for mothers from Shanghai/China and the western United States to further explore intergroup differences in predictions of parental cultural values on their parenting.

#### Correlations Between Cultural Values and Parenting

Results suggested a few similar and different patterns of associations between two ethnical groups (Table 4). For example, both groups of mothers’ cultural values were associated with parental ideologies in training and their individualistic and collectivistic parental goals were correlated.

**TABLE 4:** Correlations of measures for MC and European American mothers.

	**1**	**2**	**3**	**4**	**5**	**6**
1.Cul_COL	−	0.08	0.50**	0.53**	0.06	0.18
2.Cul_IND	0.52**	−	0.32**	0.08	0.21	–0.06
3.CRIQ	0.41**	0.12	−	0.51**	–0.08	–0.04
4.ICITCS-COL	0.35**	0.27**	0.36**	−	0.31**	0.17
5.ICITCS-IND	0.33**	0.23*	0.40**	0.84**	−	0.46**
6.PDI-Nurturance	0.24*	0.08	0.25*	0.26*	0.28**	−

*p < 0.05, **p < 0.01. Elements in lower (upper) triangular matrix are from MC (European American). Cul_COL, Collectivism subscale of cultural values; Cul_IND, Individualism subscale of cultural values; CRIQ, Child-Rearing Ideologies Questionnaire; ICITCS-COL/ICITCS-IND, Importance of Collectivis t and Individualist Traits in Child Scale; PDI-Nurturance, Nurturance dimension of Parenting Dimensions Inventory. PDI-Nurturance = Nurturance dimension of Parenting Dimensions Inventory.

In terms of different patterns, first, Shanghai mothers’ individualistic and collectivistic cultural values were moderately correlated (*r* = 0.52, *p* < 0.01) while European American mothers’ cultural values were not (*r* = 0.08, *p* > 0.05). Second, Shanghai mothers’ cultural values were correlated with their parental goals, but only collectivistic cultural values were associated their parental ideologies. However, European American mothers’ individualistic parental goals were not associated with their cultural values and parental ideologies. Third, Shanghai mothers’ collectivistic cultural values (*r* = 0.24, *p* < 0.05), child-rearing ideologies (*r* = 0.25, *p* < 0.05), collectivistic parenting goals (*r* = 0.26, *p* < 0.05), and individualistic parenting goals (*r* = 0.28, *p* < 0.01) are associated with their parenting practices. But European American mothers’ cultural values and child-rearing ideologies are not correlated to their parenting practices. Only individualistic parenting goals are associated with their parenting practices (*r* = 0.45, *p* < 0.01).

#### Interaction Effects of Ethnicity and Cultural Values

In order to examine whether mothers’ cultural values could predict their parental values and goals, and also predict their parenting practices, interaction effects between ethnicity and cultural values were examined in advance. Multiple hierarchical linear regression analyses have been conducted so that product term of ethnicity with each cultural value were entered as independent variables. Parental values, goals (i.e., individualistic and collectivistic goals), and parenting practices served as dependent variables, respectively. For each hierarchical linear regression analysis, significant interaction effects between ethnicity and collectivistic cultural values were found on parental collectivistic goals (β = 0.07, *p* = 0.001), individualistic goals (β = −0.03, *p* = 0.05), parental values (β = 0.09, *p* < 0.001), and practices (β = −0.09, *p* < 0.001). No significant interaction effects were found for ethnicity and individualistic cultural values. Therefore, the effects of cultural values on parental values, goals, and practices were examined separately for each ethnicity group.

#### Predictions of Shanghai Mothers’ Cultural Values to Parenting

According to the result of high correlation between collectivistic and individualistic cultural values for mothers from Shanghai/China, two hierarchical regression analyses were conducted to examine which cultural value was more likely to predict mothers’ child-rearing ideologies and goals. In the first analysis, two blocks of independent variables were entered into the model as predictors of mothers’ child-rearing ideologies. Collectivistic cultural values served as block one and both collectivistic and individualistic cultural values served as block two. As shown in Table 5, Shanghai mothers’ collectivistic cultural values predicted mothers’ child-rearing ideologies, *F*(1, 94) = 19.16, *p* < 0.001, and explained 17% of Mainland Chinese mothers’ child-rearing ideologies in training. In block two, Shanghai mothers’ individualistic cultural values did not predict their child-rearing ideologies.

**TABLE 5:** Hierarchical regression of Mainland Chinese mothers’ child-rearing ideologies.

**Variable**	**B**	**SE**	**β**	***t***	***R*^2^**	**Δ *R*^2^**
**Step 1**					0.17	
Collectivistic cultural values	0.37	0.08	0.41	4.38***		
**Step 2**					0.18	0.01
Collectivistic cultural values	0.42	0.10	0.48	4.37***		
Individualistic cultural values	–0.13	0.11	–0.13	–1.17		

***p < 0.001.

According to high correlation between individualistic and collectivistic parenting goals for mothers from Shanghai/China, a combined score of the two variables was used as dependent variables for the current hierarchical regression analysis. Again, two blocks of independent variables were entered into the equation as predictors of mothers’ global parental goals (combined individualistic and collectivistic goals). Collectivistic cultural values served as block one and both collectivistic and individualistic cultural values served as block two. The results revealed that Shanghai mothers’ collectivistic cultural values predicted mothers’ parenting goals, *F*(1, 94) = 13.49, *p* < 0.001, and explained 12% variance of the dependent variable (Table 6). In block two, Shanghai mothers’ individualistic cultural values did not predict their parental goals.

**TABLE 6:** Hierarchical regression of Mainland Chinese mothers’ parental goals.

**Variable**	**B**	**SE**	**β**	***T***	***R*^2^**	**Δ *R*^2^**
**Step 1**					0.12	
Collectivistic cultural values	0.42	0.11	0.35	3.67*		
**Step 2**					0.13	0.01
Collectivistic cultural values	0.36	0.13	0.30	2.66*		
Individualistic cultural values	0.14	0.15	0.11	0.94		

*p < 0.05.

A hierarchical regression analysis was conducted to examine the predictors of mothers’ parenting practices (i.e., mothers’ cultural values, parenting beliefs, and goals). For this analysis, two blocks of independent variables were entered into the model as predictors of mothers’ nurturing behaviors. Shanghai mothers’ cultural values (individualism and collectivism) served as block one and mothers’ parenting goals (collectivistic and individualistic goals) and ideologies (CRIQ) served as predictors in block two (Table 7). Results reveal that Shanghai mothers’ cultural values could predict their parenting nurturance [*F*(2, 93) = 2.64, *p* < 0.05], specifically, collectivistic cultural values could significantly explain variance in parental practices (β = 0.24, *t* = 2.08, *p* < 0.05). In block two as a whole model, Shanghai mothers’ cultural values, goals, and ideologies could predict their parenting nurturance [*F*(5, 90) = 2.26, *p* < 0.05]; however, while mothers’ cultural values were controlled, either parental goals or parental ideologies did not predict mothers’ behaviors in nurturance, respectively.

**TABLE 7:** Hierarchical regression of multiple predictors of Mainland Chinese mothers’ parenting practices.

**Variable**	**B**	**SE**	**β**	***t***	***R*^2^**	**Δ *R*^2^**
**Step 1**					0.05	
Collectivistic cultural values	0.22	0.11	0.24	2.08*		
Individualistic cultural values	–0.03	0.12	–0.03	–0.22		
**Step 2**					0.11	0.06
Collectivistic cultural values	0.13	0.12	0.14	1.09		
Individualistic cultural values	–0.04	0.12	–0.04	–0.30		
Collectivistic parenting goals	0.03	0.13	0.03	0.19		
Individualistic parenting goals	0.13	0.11	0.19	1.15		
Parental ideologies	0.11	0.13	0.10	0.85		

*p < 0.05.

#### Predictions of European American Mothers’ Cultural Values to Parenting

In the first regression analysis, individualism and collectivism subscales served as independent variables while CRIQ served as the dependent variables. The results show that mother’s cultural values could predict their ideologies in the importance of training their children, *F*(2, 75) = 18.69, *p* < 0.001, and explained 33% variance of parental ideologies, *R*^2^ = 0.33. For European American mothers, endorsement of both individualistic and collectivistic cultural values predicted CRIQ scores (β = 0.50, *t* = 5.26, *p* < 0.001 for collectivism; β = 0.29, *t* = 3.07, *p* < 0.01 for individualism).

According to results of correlation analysis, European American mothers’ cultural values were not associated with their individualistic parental goals, so only one regression analysis was performed with cultural values serving as the IVs and mothers’ parental collectivistic goals serving as the DV. Results indicated that European American mothers’ cultural values could significantly predict their parenting collectivistic goals, *F*(2, 75) = 16.05, *p* < 0.001, and explained 54% variance of the dependent variable, *R*^2^ = 0.54, Δ*R*^2^ = 0.28. The estimated regression coefficients showed that collectivistic cultural values are positively related to mothers’ collectivistic goals (β = 0.53, *t* = 5.45, *p* < 0.001) but individualistic cultural values did not contribute to European American mothers’ collectivistic goals (β = 0.11, *t* = 1.13, *p* = 0.26)^1^.

## Discussion and Conclusion

Ecological theory and previous research suggest that culture plays an important role in parenting processes (Chao, 2001). The findings from this study affirm this notion and suggest that parental beliefs and goals appear, to some extent, to be culturally organized. For example, as hypothesized, mothers from Shanghai/China and the western United States had different child-rearing ideologies and goals. Shanghai parents endorsed more collectivistic goals while European American parents endorsed more individualistic goals. Meanwhile, mothers from Shanghai/China had higher levels of Chinese traditional beliefs about training.

However, the findings from this study are different from previous studies of parenting of Chinese and American parents in some important ways. First, given that little previous research has measured parents’ cultural values explicitly, in addition, little research has studied the cultural process of parenting with Mainland Chinese parents. The present study extended previous research on cross-culture and gained new insights into the cultural process of parenting, by examining the ethnicity differences among mothers’ cultural values, parental social cognitions, and parenting practices in two contexts: Mainland Chinese and European American. Second, the results of the current study suggested that mothers from Shanghai/China were both more collectivistic and individualistic than mothers from the western United States, which is partly in contrast to that found in the previous research. Researchers have suggested that Chinese or Chinese immigrant individuals possess less of an individualistic orientation than European Americans. For example, in their examination of cultural values using a collectivistic and individualistic perspective, Oyserman et al. (2002) found that of the 20 countries sampled, Chinese and European American individuals looked the most different, with Chinese individuals reporting more collectivistic and less individualistic values. A recent study compared the East German region and Chinese region also found Chinese region tend to be more collectivist and less individualistic than East German (Schönpflug and Yan, 2013). The Asian culture has stronger collectivism as compared to individualism, in such a traditional and conformist cultural context, mothers from Shanghai/China in the present study consistently showed a higher level of collectivism. Meanwhile, they also reported more individualistic. A considerable reason may be due to social change, as Schönpflug and Yan (2013) pointed out. In China, the individualistic value orientation has also developed to meet the demands of a modernized society. Especially, Chinese mothers in the present study were from the biggest and modern city, Shanghai. These individuals live in a very urban, modernized part of China, are well educated, and are considered to be from middle-class to upper-middle-class backgrounds. As such, these mothers have frequent opportunities to interact with the modern world and know Western cultural values and conceptions of parenting through their use of the internet, their educational opportunities, exposure to diverse foods, and opportunities to meet and associate personally with those of different cultures. As a result, these Shanghai mothers more highly endorsed individualistic as well as collectivistic cultural values. Given that this sample was based largely on individuals who were not parents, it is limited in its ability to generalize to the larger population.

Second, results of the current study suggested that cultural values could be important predictors of parental ideologies and goals. However, these predictions reveal strong cultural roots of parenting issues in both ethnic groups. For example, only for Shanghai mothers, their collectivistic cultural values were related to their parental goals and ideologies beliefs, while for European American parents, their individualistic cultural values have no relationships with their individualistic goals and practices. Meanwhile, inconsistent with the hypotheses, the predictive effects of cultural values on parenting practices were not significant for European American and Shanghai mothers.

Therefore, despite the fact that mothers from Shanghai/China were higher on both individualism and collectivism, when it came to which values were most important in predicting parental social cognitions, our results showed that Shanghai mothers’ collectivistic values were more important. One explanation of this different prediction pattern for European American and Shanghai mothers could be their beliefs about important guidelines directing their parenting behaviors. Mothers from Shanghai/China believe Chinese traditional cultural values, which includes theories of education and parenting, such as the historical roots of Confucian sources (Kojima, 1986; Chao, 2000). From this perspective, children are considered to be innocent, lacking in knowledge, and have innate goodness (Boocock, 1991). One of the most widely identified characteristics of Confucian philosophies is the emphasis Chinese parents place on their children’s acquisition of academic skills (Huntstinger et al., 1997). For mothers from the western United States, their parenting goals were important contributions of their parenting behaviors. As such, their goals about self-esteem and creativity direct their behaviors of nurturance.

Several limitations of the present study need to be noted. First, our sample was a convenience sample from the biggest city of China and rural area of the United States. As a result, our findings cannot be generalized to most Mainland Chinese parents. Second, only mothers completed self-reported questionnaires. Fathers’ cultural values, parental social cognition, and parenting practices were missing. Literatures have reported different parenting styles, involvement, and practices between mothers and fathers (Cabrera et al., 2002). So, parents’ gender could be an important impact factor to parenting processes. Third, our measures were self-reported questionnaires, which may yield unreliability problems. It also should be noted that findings from the study only indicated mothers’ perception of their cultural values and parenting processes but not the “reality.” Moreover, because of the methodology issues, it was not possible to make causal inference about relationships among parents’ cultural values, parental social cognitions, and parenting practices. Future longitudinal or experimental studies are needed to explore causal relations among these parenting variables. Future studies can also incorporate acculturation experiences that can be measured by qualitative methods and explore how global process influence parenting processes.

Despite these limitations, these results suggest that parents’ cultural values are important contributors to parental beliefs and goals for their children. It makes sense that parents’ endorsement of collectivistic values should positively relate to their collectivistic goals and beliefs in the importance of training. These results fit nicely with previous research on the connection between parents’ beliefs and goals (Bornstein, 2012; Liu, 2014). In addition, the cultural roots of parenting revealed the complexities in the social dynamics worldwide. These conclusions can be used to support the positive parental cognitions and family functioning.

## Data Availability Statement

The raw data supporting the conclusions of this article will be made available by the authors, without undue reservation.

## Ethics Statement

This study was approved by the Research Ethics Committee of Washington State University, WA, United States. All participants involved in our study were given written, informed consent forms, which were signed by each participant and their guardians and returned to us. The ethics committee approved this consent procedure.

## Author Contributions

HH designed, performed, and analyzed the research, and wrote the manuscript. SU and YR revised the section of measures in manuscript. LJ searched literature and analyzed and verified the data of this manuscript. All authors contributed to the article and approved the submitted version.

## Conflict of Interest

The authors declare that the research was conducted in the absence of any commercial or financial relationships that could be construed as a potential conflict of interest.

## Footnotes

1 According to results of correlation analysis, European American mothers’ cultural values and collectivistic parental goals were not associated with their nurturance, so regression analysis was not conducted for European American mothers.
